# Diagnostic utility of transbronchial biopsy for Hodgkin's lymphoma: A case study

**DOI:** 10.1111/1759-7714.14190

**Published:** 2021-10-25

**Authors:** Miki Hoshi, Nobuaki Kobayashi, Katsushi Tanaka, Kohei Somekawa, Ayami Kaneko, Ami Izawa, Kenichi Seki, Yoichi Tagami, Ayako Aoki, Hiroaki Fujii, Keisuke Watanabe, Nobuyuki Horita, Yu Hara, Mai Matsumura, Makiko Enaka, Maki Hagihara, Takeshi Kaneko

**Affiliations:** ^1^ Department of Pulmonology Yokohama City University Graduate School of Medicine Yokohama Japan; ^2^ Department of Pathology Yokohama City University Graduate School of Medicine Yokohama Japan; ^3^ Department of Molecular Pathology Yokohama City University Graduate School of Medicine Yokohama Japan; ^4^ Department of Hematology and Clinical Immunology Yokohama City University Graduate School of Medicine Yokohama Japan

**Keywords:** biopsy, chemotherapy, lymphoma

## Abstract

Lung lesions of Hodgkin's lymphoma (HL) are rare and difficult to diagnose by nonsurgical biopsy. We herein present the case of a 72‐year‐old Japanese male who presented with accumulation of lung infiltrates and masses bilaterally on the lungs for 3 years. Although transbronchial lung biopsy (TBB) and computed tomography‐guided biopsy were conducted several times, his diagnosis remained inconclusive. On further deterioration of lung lesions, the patient was transferred to our hospital. Positron emission tomography revealed increased accumulation in the bilateral lungs and right supraclavicular lymph nodes. Surgical biopsy of the lymph node was performed. He was finally diagnosed with HL and underwent chemotherapy with doxorubicin, vinblastine, dacarbazine, and brentuximab vedotin. After chemotherapy, the lung lesion showed significant regression. A literature review indicated that the diagnostic success rate of TBB was low (18.5%) in cases of lung lesions in HL.

## Graphical abstract

Here, we report a case of undiagnosed HL with lung lesions despite multiple TBBs. Our literature review indicated that the diagnostic success rate of TBB was low (18.5%) in cases of lung lesions in HL. Surgical biopsy should be considered early to avoid a delay in diagnosis because the diagnostic ability of TBB for HL patients with lung lesions is limited.

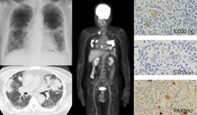

## INTRODUCTION

Lung lesions are rare in Hodgkin's lymphoma (HL) and are diagnosed based on pathological findings from samples taken during lung biopsy. Although several methods are available to obtain pathological samples for the diagnosis of HL with lung lesions, their diagnostic utilities differ. Herein, we report on a patient diagnosed with HL with lung lesions by lymph node biopsy after diagnostic failure following several attempts at transbronchial biopsy (TBB) and provide a literature review on the diagnostic utility of TBB among patients with similar clinical presentations.

## CASE REPORT

**FIGURE 1:**
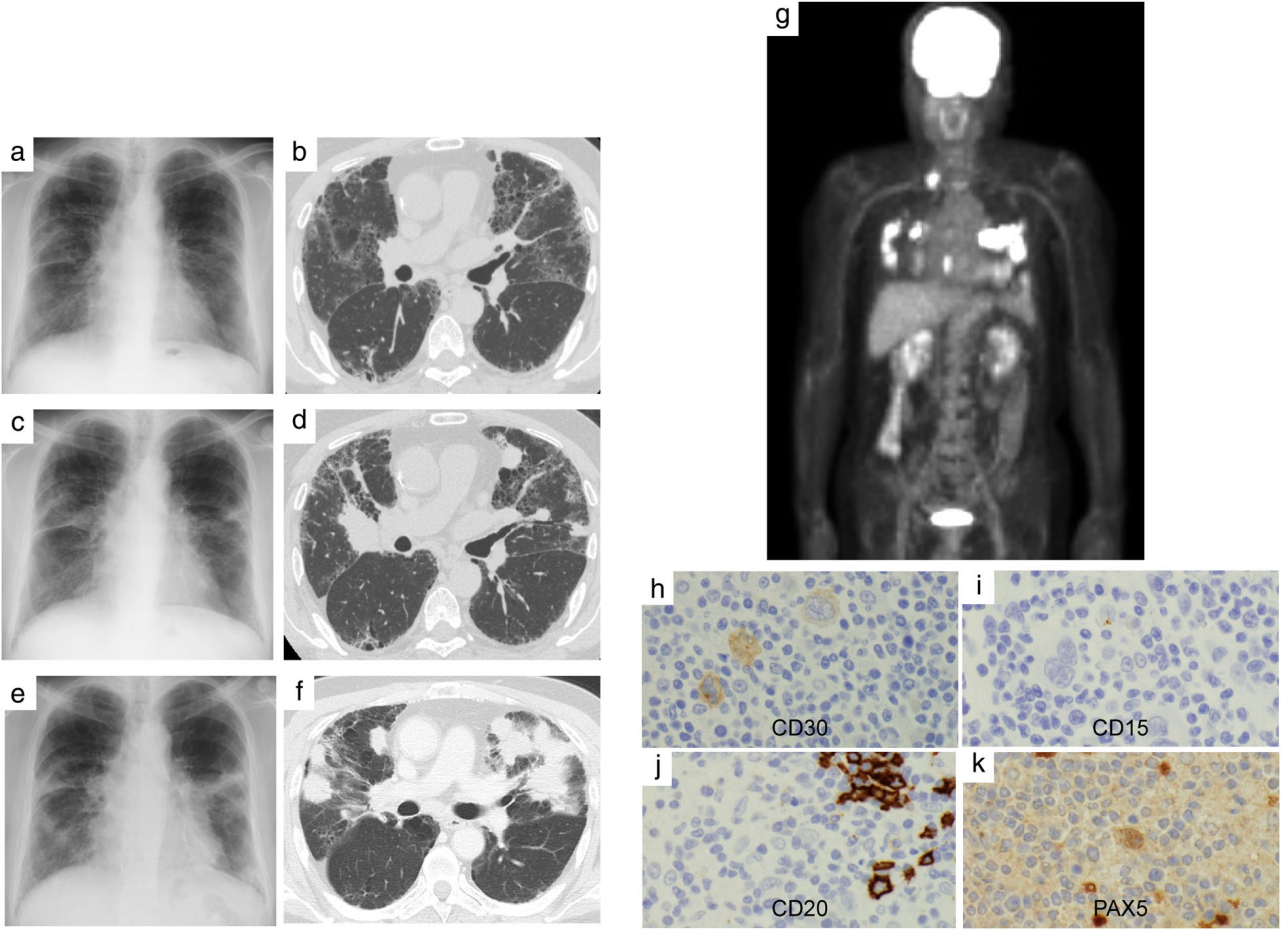
(a, b) Chest radiography and CT images obtained in 2017. Reticular and ground‐glass opacities are visible in the bilateral lungs. (c, d) Chest radiography and CT images obtained in 2018. Multiple masses are seen in the bilateral lungs. (e, f) Chest radiography and CT images obtained in 2019. Masses are enlarged and developed in consolidation. (g) Positron emission tomography performed in 2020 revealed accumulation of fluorodeoxyglucose in the lungs and right supraclavicular lymph nodes are detected. (h–k) Immunohistochemistry (IHC) of the supraclavicular lymph node biopsy. IHC is positive in CD30 (h), negative in CD15 (i), negative in CD20 (j), and positive in PAX5 (k)

A 72‐year‐old Japanese male with a smoking history of 40 cigarettes per day until 2011 was diagnosed with smoking‐related interstitial pneumonia in 2017 which was followed up with chest radiography and computed tomography (CT) (Figure 1a,b). In 2018, follow‐up radiography showed consolidation in bilateral lungs (Figure 1c,d). Although TBB and CT‐guided biopsy of the lung lesions were conducted on three occasions between 2018 and 2019, pathological findings revealed that nonspecific inflammatory cells only invaded the lung. During the TBBs, biopsy sites were confirmed with endobronchial ultrasound (EBUS) using EBUS‐guided sheath methods. On further lung involvement and deterioration, he was referred to our hospital for examination.

**FIGURE 2:**
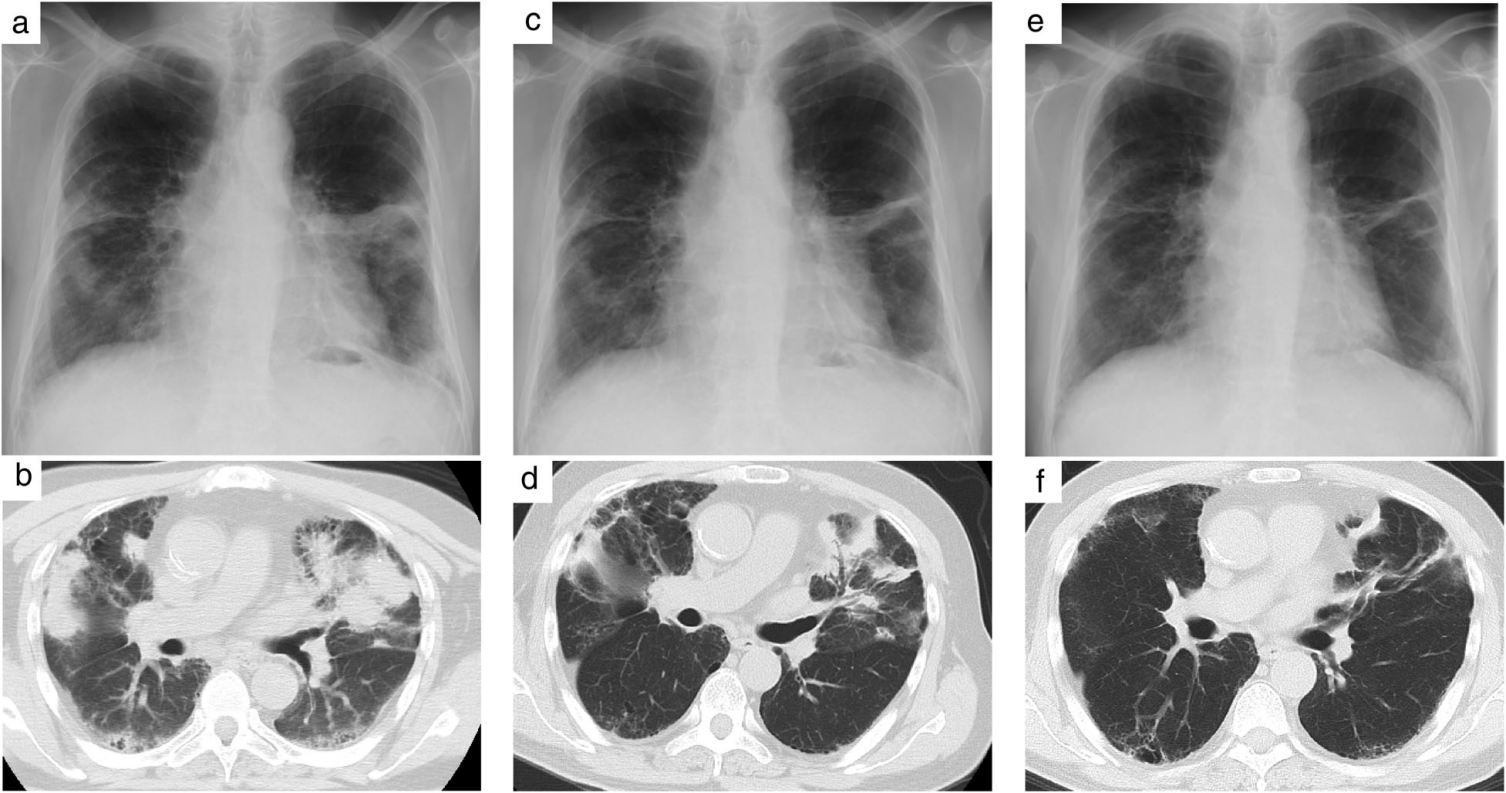
Interval changes of clinical images after chemotherapy. (a, b) Chest radiography and chest CT are performed just before the induction of chemotherapy. (c, d) Clinical images after two cycles of chemotherapy. (e, f) Clinical images after four cycles of chemotherapy

On physical examination, his vital signs and physical findings were unremarkable, respiratory rate was 16/min, SpO_2_ was 97%, and there was no palpable lymphadenopathy. Routine laboratory tests were normal, except for slightly elevated KL‐6 (783 U/ml), C‐reactive protein (8.70 mg/l), and soluble interleukin‐2 receptor (798 U/ml) levels. Lung cancer‐related tumor markers were negative. Chest radiography and CT revealed accumulation of multiple nodules in bilateral lungs (Figure 1e,f). Positron emission tomography(PET)‐CT revealed increased fluorodeoxyglucose accumulation in the right supraclavicular lymph nodes and bilateral lung lesions (Figure 1g). Surgical biopsy of these lymph nodes showed an eosinophilic granulomatous region with scattered binuclear large cells between follicles. Immunohistochemistry (IHC) was positive for cluster of differentiation (CD) 30 (Figure 1h) and negative for CD15 (Figure 1i), organic anion transporter (Oct) 1, B‐cell Oct‐binding protein (BOB) 1, and programmed cell death protein (PD) 1. CD20 and paired box 5 (PAX5) was dim. Typically, Epstein–Barr virus‐encoded small RNA (EBER) is positive in large hodgkinoid cells; however, EBER was positive only in the small cells in this patient. Although it is atypical for Hodgkin's lymphoma to have numerous CD20+ cells (Figure 1j) and negative PD‐1 in all cells, the positive IHC in PAX5 (Figure 1k) and CD30 and negative IHC in BOB‐1 and Oct‐2 were consistent with the features of Hodgkin's cells. Based on these findings, the patient was diagnosed with HL following careful pathological examination and subsequently underwent treatment with doxorubicin, vinblastine, dacarbazine, and brentuximab vedotin (A + AVD)‐based chemotherapy. Although peripheral neuropathy and febrile neutropenia emerged as chemotherapy‐related adverse events and the amount of therapy had to be reduced, six courses were completed. Following treatment, lesions on both the lungs and lymph nodes had significantly regressed (Figure 2a–f).

To explore the diagnostic utility of several biopsy methods for HL with lung lesions, a literature review was performed. A database search identified 101 cases between 1990 and 2020 (Table 1).^1^, ^2^, ^3^, ^4^, ^5^, ^6^, ^7^, ^8^, ^9^, ^10^, ^11^, ^12^, ^13^, ^14^, ^15^, ^16^, ^17^, ^18^, ^19^, ^20^, ^21^, ^22^, ^23^, ^24^, ^25^ Most cases were diagnosed by surgical biopsy (85 cases), followed by CT‐guided biopsy (eight cases) and TBB (four cases).

**TABLE 1:** Summary of lung biopsy used for the diagnosis of Hodgkin's lymphoma with lung lesions

Reference	Year	Number of cases	Biopsy method for final diagnosis			
			Transbronchial biopsy	CT‐guided biopsy	Surgical biopsy	Others
Radin^a^	1990^1^	47	1	0	46	
Schee et al.	1990^2^	2	0	0	2	
Pinson et al.	1992^3^	1	0 (1)	0	1	
Chetty et al.	1995^4^	3	0	0	3	
Cartier et al.	1999^5^	3	1 (1)	1	1	
Boshnakova et al.	2000^6^	2	0 (2)	0	2	
Stachura et al.	2003^7^	1	0 (1)	0	1	
Hosoi et al.	2005^8^	1	0	1	0	
Rodriguez et al.	2006^9^	5	1	0	4	
Codrich et al	2006^10^	1	0 (1)	0	1	
Urasinski et al.	2010^11^	3	0 (1)	0	3	
Honda et al.	2013^12^	1	0 (1)	0	1	
Cooksley et al.^b^	2014^13^	15	0 (8)	0 (3)	15	
Schild et al.	2014^14^	1	0	1	0	
Tanveer	2015^15^	1	0	0	1	
Watanabe et al.	2015^16^	1	0	1	0	
El Hage et al.	2017^17^	1	0 (1)	1	0	
Lowenthal et al.	2017^18^	2	2	0	0	
Sinha et al.	2017^19^	1	0	1	0	
Tahara et al.	2017^20^	2	0 (2)	1	1	
Aljehani et al.	2018^21^	3	0 (1)	2 (1)	1	
Conti et al.	2018^22^	1	0 (1)	1	0	
Chowdhary et al.	2020^23^	1	0	0	1	
Parente et al.	2020^24^	1	0	0	0	US‐guided aspiration: 1
Takumi et al.	2020^25^	1	0 (1)	0	1	

*Note*: ( ): Biopsy was performed but undiagnostic.

^a^ Radin reported 61 cases, but only 47 of them underwent lung biopsy evaluation.

^b^ Cooksley reported 20 cases, but two of them were diagnosed by lymph node biopsy and two of them did not mention the biopsy method, and one case was not identified.

Based on our case and the literature review, TBB and CT‐guided biopsies seem less effective for diagnosing HL with lung lesions than surgical biopsy; therefore, the diagnostic utility of those methods was analyzed using data from Table 1. The success rate for arriving at the final diagnosis via TBB was only 18.5%, which was higher for CT‐guided biopsy (71.4%).

## DISCUSSION

Here, we report a case of undiagnosed HL with lung lesions despite multiple TBBs. HL is a relatively rare malignant lymphoma (ML) in Japan (5%–10% of ML cases).^26^ Furthermore, lung parenchyma involvement in HL is uncommon and difficult to diagnose via TBB.^27^ Yao et al. reported that the median duration to arrive at the final diagnosis was 6 months and the longest duration was 2 years in 19 cases of HL with lung lesions.^28^ Smoking increases the risk of HL and may contribute to the development of cancer cells, as in our case study.^29^ Our literature review revealed that TBB is inappropriate for most cases of HL with lung lesions.

Reasons for the poor diagnostic performance of TBB and CT‐guided biopsy might be the small volume of samples obtained by both methods. The presence of Hodgkin's or Reed‐Sternberg cells in the specimen is a histological feature of HL, but these cells are few and scattered in the background of small lymphocyte infiltration. Kogawara et al. diagnosed 67% of diffuse large B cell lymphoma (DLBCL) and 33% of mucosa‐associated lymphoid tissue lymphoma by TBB or endobronchial biopsy, which is higher than the diagnostic utility of TBB for HL.^30^ Although it is more invasive for patients, with the possibility of complications especially for patients with interstitial lung disease, a surgical biopsy can solve these issues, especially in HL cases.^31^

As a tool to overcome this dilemma, transbronchial cryobiopsy (TBCB) has recently gained attention. It is transbronchial and less invasive than the surgical approach and can obtain a sample large enough to diagnose at the first instance (maximum diameter: 5–7 mm).^32^ Its usefulness in diagnosing ML has been previously reported. Bianchi et al. reported that in 12 of 13 cases of ML, diagnosis was reached via TBCB, with two cases being HL. Dante et al. diagnosed DLBCL via TBCB after failure with TBB. Successfully diagnosed cases of TBCB in HL are few and TBCB is unavailable at most facilities. Our case had a history of smoking‐related interstitial pneumonia with concerns regarding exacerbation of respiratory function; therefore, a surgical biopsy was avoided for many years. One cause of HL is smoking. Cancer cells can migrate to the lungs as a result of smoking. Imaging features of HL of the lung include solitary or multiple nodules and alveolar consolidation.^33^ There is also predilection of the upper lobes in cases of primary pulmonary HL.^15^, ^27^ Differential diagnosis of HL includes lung cancer, metastasis, lymphocytic interstitial pneumonia, and cryptogenic organizing pneumonia. In our case, chest CT showed multiple nodules and consolidation in bilateral lungs and TBB was not diagnostic. Thus, we could not rule out HL and decided to perform surgical biopsy of the right supraclavicular lymph nodes. This case study demonstrates that surgical biopsy should be conducted for suspicious lesions.

In conclusion, TBB or CT‐guided biopsy remains a common diagnostic tool for HL patients with lung lesions, but its diagnostic ability is limited. Surgical biopsy or TBCB should be considered early to avoid a delay in diagnosis.

## CONFLICT OF INTEREST

On behalf of the co‐authors, the corresponding author declares no conflicts of interest.
